# Evaluating and Improving the Quality of Surgical Operative Notes at the Port Sudan Teaching Hospital

**DOI:** 10.7759/cureus.75815

**Published:** 2024-12-16

**Authors:** Alnazeer Y Abdelbagi, Abubakr Muhammed, Mohey Aldien Ahmed Elamin Elnour, Mustafa Mohamed, Amir Alamin Ahmed Altyeb, Alaa Osman Sir Alkhatim Ali, Mohamed Khalid Hassan Abdelkarim, Fatima Aladdin Elssayed Osman, Elbokhari Mohamed Elbashir Hassan, Mahmoud Ali Mohamed Abdalrahman, Galaleldin Mohamed Abdeljalil Mohamed, Etigad Salahuddin Elzubier Suliman, Khalid Ahmed Noureldaim Ahmed, Awad Abdalla Widaa Mohamed

**Affiliations:** 1 Anatomy, University of Khartoum, Khartoum, SDN; 2 Surgery, University of Gezira, Madani, SDN; 3 General Surgery, Port Sudan Teaching Hospital, Port Sudan, SDN; 4 Internal Medicine, AL-Neelain University, Khartoum, SDN; 5 General Medicine, Prince Sultan Military Medical City, Riyadh, SAU

**Keywords:** audit cycle, general surgery, operative notes, quality improvement, royal college of surgeons

## Abstract

Background

Thorough and standardized documentation of operative notes is essential for effective communication, patient safety, legal protection, and the continuity of care. However, in many hospitals in Sudan, surgeons often use non-standardized methods, resulting in inconsistent and incomplete records. This study evaluates the quality of operative notes at the Port Sudan Teaching Hospital using the Royal College of Surgeons of England (RCSEng) guidelines, aiming to improve compliance and documentation practices.

Methods

A clinical audit was conducted in two cycles at the General Surgery Department. The first cycle, a retrospective review of 50 surgical notes, was carried out over one week in July 2024. The second cycle conducted prospectively on another 50 notes throughout September 2024, involved the implementation of an improved proforma and staff training. Data were collected using a standardized checklist aligned with Royal College of Surgeons in Ireland (RCSI) guidelines, covering 18 documentation criteria. Results were analyzed using Microsoft Excel 2016 (Microsoft Corporation, Redmond, Washington, United States) to assess improvements in compliance.

Results

The compliance with documentation standards increased significantly from 51.9% in the first cycle to 82.1% in the second cycle. Notable improvements were observed in recording operative findings (17, 34%), complications (34, 68%), and deep vein thrombosis (DVT) prophylaxis (47, 94%). Despite the overall progress, modest improvements were noted in documenting the anesthetist's name (2.5, 5%) and the surgeon’s signature (3, 6%). These findings underscore the positive impact of structured proformas and targeted staff training.

Conclusion

The implementation of standardized documentation tools and staff training significantly improved the quality of surgical operative notes at the Port Sudan Teaching Hospital. While notable progress was achieved, continued efforts, including digital solutions and regular audits, are needed to sustain these improvements and promote patient safety.

## Introduction

Thorough and precise operative reports are indispensable in the field of surgery. These reports, which document the specifics of each surgical procedure and its outcomes, are foundational to maintaining accurate patient records. Regardless of the type of surgery or clinical environment, operative reports serve multiple critical functions. They facilitate communication between healthcare providers, including referring and consulting physicians. Additionally, these reports are essential for the training and development of medical residents, supporting both educational and research activities. They also provide crucial documentation in legal contexts, such as malpractice claims, and ensure that billing is compliant with procedural requirements. In essence, operative reports are a cornerstone of both clinical practice and the broader healthcare system, supporting patient care, legal integrity, and administrative processes [1].

The significance of the thoroughness of operative reports and their potential impact on patient outcomes is only beginning to be explored. Recently published studies examining a group of patients highlighted that the failure to properly document the dissection of Calot's triangle was linked to adverse outcomes, specifically common bile duct injury during cholecystectomy. This finding underscores the importance of including critical details in operative reports, as it suggests that documenting essential elements could enhance patient safety and serve as valuable educational markers for surgeons in training [2].

Unfortunately, another situation where the significance of an incomplete operative report becomes particularly apparent is in medical malpractice litigation. In such cases, a detailed and well-organized operative report can serve as key evidence of a surgeon's competence, while an incomplete report, though not direct evidence of negligence, may be used to suggest substandard care and poor documentation practices. This underscores the need to consider not only the limited studies on the structure of operative reports but also the overall quality and completeness of these reports in clinical practice [2,3].

Documentation in operative notes is no exception to the rigorous standards required in medical practice. The Royal College of Surgeons of England (2014) has developed comprehensive guidelines titled Good Surgical Practice, which set forth the minimum documentation standards expected in surgical practice. These guidelines emphasize the critical role that accurate and thorough documentation plays in ensuring patient safety, continuity of care, and effective communication within the healthcare team. According to these guidelines, operative notes should provide a clear, concise, and complete record of the surgical procedure [4].

In numerous hospitals across Sudan, surgeons often resort to using blank paper for documenting operative notes. This practice leads to inconsistent and incomplete records, which can have serious repercussions. The absence of standardized templates may result in critical omissions that compromise both patient care and the legal integrity of medical records. By adopting structured templates, as recommended by the Royal College of Surgeons of England (RCSEng), we could significantly improve the quality and consistency of surgical documentation in these settings. Such implementation would ensure more reliable and comprehensive records, ultimately enhancing patient outcomes and safeguarding against legal challenges [5].

## Materials and methods

This retrospective prospective study aimed to evaluate the quality of operative notes under the Royal College of Surgeons of England (RCSEng) Guidelines (Table 1). Conducted at the Port Sudan Teaching Hospital in Port Sudan, Red Sea State, the study covered the period from the 26th of July 2024 to the 20th of September 2024. The primary goal was to determine whether the operative notes adhered to the RCS standards (Table 1), which emphasize accuracy, comprehensiveness, and clarity. Ensuring compliance with these guidelines is crucial for improving the efficiency of surgical teams and the overall quality of patient care [6]. The Institutional Review Board of the Port Sudan Teaching Hospital granted ethical approval for this study.

**Table 1:** The standards of the Royal College of Surgeons of England [6]

Parameters Checklist:
Date and time
Type of operation
Name of operator and assistant
Name of anesthetist
Operative procedure carried out
Incision type
Operative diagnosis
Operative finding
Problems/complications
Any extra procedures and why it was performed
Details of tissue removed, added or altered
Identification of prostheses/material used
Details of closure technique
Anticipated blood loss
Prophylaxis antibiotics
Deep vein thrombosis (DVT) prophylaxis
Details of postoperative care instructions
Surgeon’s signature

Aim and objectives

Our aim is to enhance patient safety and optimize surgical procedures through the implementation of standardized and consistent surgical operative notes. By developing comprehensive recommendations and interventions, we seek to elevate the quality of these notes, specifying essential fields, proper documentation practices, and any special requirements. Additionally, provide training sessions for healthcare professionals to ensure the effective use of these standardized notes.

Audit area/population

At the Port Sudan Teaching Hospital, a detailed audit was carried out to evaluate the quality of surgical operative notes in the General Surgery Department. This audit involved two distinct cycles: the first cycle, conducted retrospectively for one week in July 2024, and the second cycle, carried out prospectively throughout September 2024. The primary goal of this audit was to assess and ensure compliance with the RCS standards for documenting surgical operation details, thereby aiming to enhance the overall quality and consistency of patient records and care within the department.

Sample size and sampling technique

In this study, 100 surgical operative notes were analyzed from the General Surgery Department. This analysis was divided into two parts: 50 notes were reviewed retrospectively over one week in July 2024, and another 50 notes were assessed prospectively in September 2024. We employed a simple random sampling technique to ensure the selected notes accurately represented the broader spectrum of cases managed by the department. This approach encompassed a diverse range of operations, thereby ensuring a comprehensive evaluation.

Data collection method and analysis

At the Port Sudan Teaching Hospital, skilled medical staff meticulously gathered data by reviewing surgical operative notes using a standardized checklist aligned with RCS guidelines. The checklist covered 18 specific criteria, focusing on the accuracy and completeness of documentation. In the first phase, conducted retrospectively over one week in July 2024, 50 randomly selected notes were analyzed. The second phase, conducted prospectively throughout September 2024, involved improved proforma and staff training to ensure proper documentation practices. The collected data was then analyzed using Microsoft Excel 2016 (Microsoft Corporation, Redmond, Washington, United States) to measure enhancements in documentation quality between the two phases.

## Results

The analysis conducted at the Port Sudan Teaching Hospital revealed significant improvements in the documentation quality of surgical operative notes across two audit cycles. During the initial cycle, conducted retrospectively over one week in July 2024, and the second cycle, performed prospectively throughout September 2024, data showed marked enhancements in various documentation criteria following the implementation of an improved proforma and staff training.

The documentation of the date and time of surgeries improved from 37 (74%) in the first cycle to 47 (94%) in the second cycle. The type of operation recorded increased from 40 (80%) to 49 (98%). The inclusion of the names of the operator and assistant rose from 41 (82%) to 49 (98%), while the documentation of the anesthetist's name improved from 28 (56%) to 31 (61%). The accuracy of recording the operative procedure carried out increased from 42 (84%) to 49 (98%).

The documentation of the incision type improved from 31 (62%) to 43 (86%), and the operative diagnosis rose from 33 (66%) to 45 (90%). The recording of operative findings increased from 33 (66%) to 50 (100%). The documentation of problems or complications encountered during surgery improved from 15 (30%) to 49 (98%). The documentation of any extra procedures performed and their reasons rose from three (6%) to 47 (94%).

The recording of details of tissue removed, added, or altered improved from 17 (34%) to 40 (80%). The identification of prostheses or materials used increased from seven (14%) to 38 (76%). The documentation of the closure technique rose from 25 (50%) to 48 (96%). The recording of anticipated blood loss improved from five (10%) to 46 (92%), and the documentation of prophylaxis antibiotics rose from 18 (36%) to 48 (96%).

The documentation of deep vein thrombosis (DVT) prophylaxis increased from two (4%) to 49 (98%). Details of postoperative care instructions rose from 40 (80%) to 49 (98%). Finally, the surgeon’s signature documentation improved from 36 (72%) to 39 (78%).

Overall, these findings demonstrate substantial enhancements in the quality of surgical documentation at the Port Sudan Teaching Hospital. The improvement in documentation between the first and second cycle is illustrated in Table 2.

**Table 2:** The compliance rates for documentation in the first and second cycles were reported, along with the percentage of improvement.

Criteria	First Cycle	Second Cycle	Improvement (%)
Date and time	37(74%)	47(94%)	20%
Type of operation	40(80%)	49(98%)	18%
Name of operator and assistant	41(82%)	49(98%)	16%
Name of anesthetist	28(56%)	31(61%)	5%
Operative procedure carried out	42(84%)	49(98%)	14%
Incision type	31(62%)	43(86%)	24%
Operative diagnosis	33(66%)	45(90%)	24%
Operative finding	33(66%)	50(100%)	34%
Problems/complications	15(30%)	49(98%)	68%
Any extra procedures and why performed	3(6%)	47(94%)	88%
Details of tissue removed, added, altered	17(34%)	40(80%)	46%
Identification of prostheses/material used	7(14%)	38(76%)	62%
Details of closure technique	25(50%)	48(96%)	46%
Anticipated blood loss	5(10%)	46(92%)	82%
Prophylaxis antibiotics	18(36%)	48(96%)	60%
Deep vein thrombosis (DVT) prophylaxis	2(4%)	49(98%)	94%
Details of postoperative care instructions	40(80%)	49(98%)	18%
Surgeon’s signature	36(72%)	39(78%)	6%

## Discussion

The study demonstrates a significant improvement in medical documentation at the Port Sudan Teaching Hospital, with compliance rising from 51.9% in the first audit cycle to 82.1% in the second. This notable progress reflects the effectiveness of the interventions applied and underscores their broader impact on enhancing patient care, clinical practices, and healthcare system efficiency.

Another study done in Dongola Teaching Hospital, Sudan, demonstrated an increase in compliance with Good Surgical Practice guidelines from 50.3% in the initial audit cycle to 71.9% in the second [7], with notable improvements in recording additional procedures and prosthesis serial numbers. These enhancements underscore the critical role of standardized documentation in improving the accuracy and completeness of surgical records. Together, these studies highlight the importance of structured practices in promoting patient safety, ensuring continuity of care, and meeting professional documentation standards, reinforcing the value of implementing such systems across healthcare settings. The similarity in results between the two studies can be attributed to the shared working environment and comparable contextual factors within the Sudanese healthcare system.

Proper surgical operative note documentation is essential for high-quality patient care, effective information transfer, and legal protection for healthcare professionals. A clinical audit at Jordan University Hospital using the Surgical Tool for Auditing Records (STAR) demonstrated significant improvements in documentation quality between 2016 and 2021, driven by the adoption of electronic medical records, regular audits, and staff education. These improvements highlight the role of proper documentation in enhancing patient management, care continuity, and adherence to clinical standards, ultimately improving patient outcomes [5].

In global comparisons, Singh et al. (2012) reported initial deficiencies in surgical documentation, which were significantly improved through educational interventions and memory aids to nearly 100% compliance. While our audit also showed notable progress, achieving similar levels of compliance may require additional strategies like checklists or reminders [8].

Mulwafu (2018) in Malawi highlighted significant challenges in surgical documentation, particularly in recording details such as the time of operation, urgency, and estimated blood loss, with some areas showing compliance rates as low as 0%. Following targeted interventions, there was a notable improvement, with most variables achieving compliance rates above 60%. However, despite these advancements, issues with completeness and legibility persisted [9]. These findings mirror trends in our study, where substantial progress was made in documenting complications and additional procedures, but difficulties remained in ensuring the completeness of postoperative care instructions and maintaining clear, legible records.

In Nigeria, Babalola et al. (2016) observed significant shortcomings in the quality of surgical documentation, with only 37% of operation notes meeting appropriate standards. The study identified poor legibility and incomplete entries as primary contributors to these deficiencies [10]. Similar patterns emerged in our findings, particularly in legibility and the thoroughness of postoperative care instructions. This similarity underscores the widespread nature of these challenges and highlights the pressing need for targeted interventions to improve documentation practices across diverse healthcare systems.

Rogers (2008) found that consultants in South Africa were generally more diligent in recording surgical details but often neglected critical aspects such as postoperative orders and wound closure techniques [11]. While our study did not distinguish between consultants and trainees, it similarly revealed shortcomings in documenting postoperative instructions. These parallels emphasize the importance of implementing standardized documentation practices to ensure consistency and thoroughness across all levels of surgical personnel.

Cutting et al. (2014) in the United Kingdom demonstrated that conducting an initial audit followed by a re-audit led to substantial improvements in the completeness of operation notes, particularly in recording operative findings, where compliance increased from 94% to 100% [12]. Similarly, our study showed progress in this area, although issues with documenting postoperative care instructions persist. The success of structured proformas in Cutting’s study indicates that their broader application could effectively address these gaps in our context.

In their 2020 study, Javid et al. in India demonstrated substantial improvements in documentation quality following a closed-loop audit, with compliance rates surpassing 96% for most variables in the second cycle [13]. While our study also achieved significant progress, especially in documenting DVT prophylaxis and complications, we did not reach the same level of compliance. The higher compliance observed in Javid's study may be partly attributed to the implementation of electronic health records, suggesting that integrating such digital tools could be advantageous in our setting to further improve documentation accuracy.

Hassan et al. (2023) in Egypt found that regular audits and feedback contributed to a significant increase in compliance with operative record documentation, with rates rising from 86.15% to 95.6% after the interventions [14]. In contrast, our study, while showing progress, did not reach such high levels of compliance. The results from Hassan’s study suggest that the combination of audits and feedback can be a powerful method for maintaining high documentation standards. Adopting a similar approach in our system might further boost the consistency and quality of our documentation practices.

In our opinion, the significant improvements in surgical documentation observed in this study are largely attributable to the structured interventions implemented, including the use of an enhanced proforma and targeted staff training. These strategies likely contributed to the marked increase in the documentation of critical surgical details such as the date and time of surgery, type of operation, and complications encountered. However, there are areas, such as the documentation of the anesthetist's name and the surgeon’s signature, where the improvements were less pronounced. This may reflect habitual documentation practices that are more difficult to change and may require further targeted efforts.

One potential reason for the relatively modest improvement in certain areas could be the persistence of traditional documentation habits. In many cases, healthcare professionals may be accustomed to recording certain details outside the standardized surgical notes. For instance, the anesthetist’s name may be documented separately in anesthesia records, creating a gap in the completeness of surgical notes. To address this, it is essential to emphasize the importance of integrating all relevant information into the surgical notes themselves. This could be achieved through additional training sessions, reinforcement during audits, and the inclusion of reminders in the proforma templates.

The significant increase in the documentation of operative findings, complications, and additional procedures points to a heightened awareness of the need for thorough documentation in these areas, which is crucial for patient safety and continuity of care. However, the study also highlights the challenges in achieving uniform compliance across all documentation criteria. Despite improvements in most areas, the documentation of postoperative care instructions and the surgeon’s signature still shows some room for further enhancement. These findings are consistent with other studies, which suggest that certain documentation components, such as postoperative instructions, continue to be problematic in many clinical settings.

By addressing deficiencies in the documentation process and ensuring adherence to standardized criteria, the study sought to enhance the accuracy, consistency, and completeness of surgical records. The study successfully achieved its aim and objective, as evidenced by the significant improvements observed in the documentation of surgical operative notes across the two audit cycles.

Limitations and recommendations

Ongoing auditing and feedback systems will be crucial to maintaining the improvements achieved in this study. Moreover, implementing digital tools, such as electronic medical records (EMRs), could help simplify the documentation process and enhance compliance. While the study's findings are encouraging, its limitations, such as the relatively short timeframe and its focus on a single hospital may restrict their applicability to other healthcare settings. Consequently, further studies with larger sample sizes and across multiple centers are needed to evaluate the wider relevance of these results.

## Conclusions

The study reveals significant improvements in surgical documentation at the Port Sudan Teaching Hospital after introducing targeted interventions. However, achieving consistent compliance remains a challenge, especially in documenting postoperative care instructions and signatures. The findings highlight the importance of structured approaches, such as proformas and training, in enhancing documentation standards and supporting patient safety.
